# Biomolecule-Based Biomaterials and Their Application in Drug Delivery Systems

**DOI:** 10.3390/ijms24076425

**Published:** 2023-03-29

**Authors:** Helena P. Felgueiras

**Affiliations:** Centre for Textile Science and Technology, University of Minho, Campus de Azurém, 4800-058 Guimarães, Portugal; helena.felgueiras@2c2t.uminho.pt

The antibiotic crisis is a global concern. The increasing rates of microbial resistance to antibiotics have led researchers to question their efficiency and to invest their efforts into other molecules, particularly those of natural origin. Indeed, Chittasupho et al. examined the combined effects of quercetin and curcuminoid extracts on the antimicrobial, antioxidant, and cell migration activities, and overall wound healing properties, and assessed their potential for applications in wound therapies. They determined that these extracts exhibit synergistic effects against bacteria, that they require lower concentrations to ensure the same antioxidant effects than the single extracts, and that at a 3:1 ratio of quercetin/curcuminoid, the combination is non-toxic against cells and can instigate cell migration and wound closure, thus evidencing their therapeutic features [1]. Biomolecules derived from natural extracts are not new in medicinal formulations. In fact, they have been used for many years in traditional medicine and even in cosmetic and fragrance formulations with antioxidant, analgesic, relaxing, and other therapeutic abilities. In odorant formulations, supramolecular gels are a novelty to control the release of fragrances via peptide-based low-molecular-weight molecules that are completely biocompatible and able to self-assemble into long fibers [2]. They are gaining a greater presence and impact because of their ecological features, lower environment impact, renewable sources, and, most importantly, their inherent antimicrobial and therapeutic potential. Among these, antimicrobial peptides can be emphasized for their broad spectrum of antimicrobial activity against a wide range of pathogens with low immunogenicity against mammalian host cells. However, all biomolecules present fragility and sensitivity to external factors (temperature, pH, etc.), limiting their broad, unprotected use. Drug delivery systems generated from new biomaterial formulations or new intricate, complex scaffolding systems based on nano- to microsystems or hydrogel-based constructs have arisen as the solutions to these problems. New functionalization strategies have also been explored, such as by means of primary, secondary, or tertiary coatings, namely, binding sequences, layers, or loading into matrices, using a wide variety of binding and/or protection agents [3]. Additionally, different biomaterials of synthetic and natural origins have been used in the production of such systems and devices, protecting the payload and regulating its release. Among the many alternatives, the natural polymer silk fibroin, the organic matter obtained from the cocoons of a silkworm *Bombyx mori* that has frequently been associated with textile formulations, has piqued the interest of the biomedical community as a possible biopolymer for such drug delivery applications due to its high biocompatibility, physicochemical and mechanical stabilities, and versatility, reporting outstanding results [4]. 

Currently, trigger-based release biomolecule-loaded systems represent a significant portion of the drug loading formulations under study. From natural extracts to traditional medicine remedies and newly engineered delivery platforms, many formulations that combine one or more bioactive molecules are being developed, with the purpose of fighting infections or treating specific illnesses in a localized, specialized manner. Indeed, Curcio et al. engineered a target-directed, trigger-based lipid–polymer nanoparticle to work as a delivery vehicle of doxorubicin hydrochloride that actively targeted CD44-receptors and responded to dual stimuli (pH/redox). They report the efficiency of the spherical nanoparticles to be internalized by the cancer cells through a receptor-mediated endocytosis process, and to modulate the release of the entrapped drug via the media pH and redox potential conditions. Most importantly, the smart multifunctional nanoparticles were found to be safe towards human cells, being deemed as a potential alternative for cancer treatment [5]. Following in these footsteps, Noreen et al. also proposed a pH-responsive system for anti-cancer drug delivery therapies. Here, methotrexate, a potent folic acid antagonist, was encapsulated within nanoparticles prepared from *Abelmoschus esculentus* mucilage and chitosan by a coacervation method. Nanoparticles were characterized as spherical in shape and pH-dependent, guaranteeing the controlled liberation of the entrapped drug over a period of 32 h. In vivo toxicity examinations attested to the efficiency of the engineered methotrexate-loaded nanoparticles in eliminating tumoral cells, thus confirming their therapeutic potential [6]. 
